# Enrichment Extraction and Activity Study of the Different Varieties of *Hericium erinaceus* against HCT-8 Colon Cancer Cells

**DOI:** 10.3390/molecules28176288

**Published:** 2023-08-28

**Authors:** Zhongrui Cao, Zhijun Zhang, Dongsheng Wei, Mingyu Guo, Shufang Li, Hanyuan Cui, Yue Zhang, Yuwei Zhang, Xiaoming Chen

**Affiliations:** 1 Institute of Agro-Products Preservation and Processing Technology, Tianjin Academy of Agricultural Sciences, Tianjin 300380, China; zhongruicao@163.com (Z.C.);; 2 Department of Microbiology, College of Life Sciences, Nankai University, Tianjin 300071, China

**Keywords:** *Hericium erinaceus*, supercritical CO_2_ extraction, HCT-8 colon cancer cells, GC-MS

## Abstract

*Hericium erinaceus* (HE), a widely utilized natural remedy and dietary source, has garnered significant attention for its therapeutic potential in various diseases. In this study, we employed supercritical fluid extraction (SFE) technology to isolate the bioactive compounds from HE’s fruiting body. Comprehensive assessments of the antioxidant and antibacterial activities were conducted, along with in vitro investigations on the human colon cancer cell line (HCT-8). The SFE rate served as the evaluation metric, while the variables of extraction time, pressure, and temperature were systematically examined. By integrating the response surface center composite design, we successfully optimized the extraction process, yielding optimal parameters of 80 min, 30 MPa, and 35 °C, thus resulting in an extraction rate of 2.51%. These optimized conditions exhibited considerable antioxidant capacity, anticancer activity, and antibacterial potential. Furthermore, we employed graded alcohol extraction to refine the crude extracts, thereby confirming superior anticancer effects under a 70% alcohol precipitation. To elucidate the composition, Fourier-transform infrared spectroscopy (FT-IR) and gas chromatography-mass spectrometry (GC-MS) were employed to analyze the crude extracts and isolates of HE, facilitating a comparative analysis of six HE varieties. Our findings suggest that sterol derivatives hold promise as the active component against the colon cancer HCT-8 cell line. In conclusion, this study underscores the potential of HE SFE in the development of functional foods or alternative drugs for colon cancer treatment, thus opening new avenues for therapeutic interventions.

## 1. Introduction

*Hericium erinaceus* (HE), known by various names such as Monkey Head mushroom, Lion’s Mane, Houtou (Chinese), and *Yamabushitake* (Japanese), is a highly versatile mushroom with both culinary and medicinal uses [1,2]. Traditional Chinese medicine has long recognized the value of HE, leading to its widespread application in various therapeutic contexts. Recent pharmacological investigations have unveiled the presence of polysaccharides, triterpenes, fatty acids, and other bioactive compounds in HE, highlighting its potential for anti-ulcer, anti-inflammatory, and immune modulation effects, as well as blood sugar and lipid regulation [3,4,5,6,7,8]. Consequently, the extraction of these valuable components from HE has become a focal point for research and development [9]. While solvent extraction has been conventionally employed [10], there is growing interest in SFE as an alternative approach due to its solvent-free nature and minimal thermal degradation [11]. Supercritical fluid extraction (SFE), as it utilizes the unique properties of supercritical CO_2_ fluid, surpasses traditional solvent extraction methods by enhancing mass transfer efficiency, eliminating solvent residues, offering operational convenience, controlling pollution, reducing toxicity, and ensuring product purity [12]. Particularly, SFE holds promise for the extraction of active components from HE, thus paving the way for large-scale industrial production and practical applications in various fields.

According to the latest cancer data released by the WHO Cancer Research Agency (IRAC) in 2020, colorectal cancer is the most common malignant tumor of the digestive tract in the world, second only to breast cancer and lung cancer [13]. Since 2018, about 1.8 million new cases of colorectal cancer have been reported [14], and this is expected to increase to about 22.1 million cases by 2030 [15]. However, the most serious problem encountered by patients with colorectal cancer is the increased resistance of cancer cells to mainstream chemotherapy drugs, as well as the side effects of cytotoxic drugs [16], which can lead to drug-induced hepatotoxicity. Therefore, the extraction and development of the potential bioactive components in natural products such as alternative drugs or functional foods [17] as well as the evaluation of their mechanisms of action are crucial for the development of colorectal cancer replacement therapy.

Building upon the discovery of the inhibitory effect of the SFE from HE on colon cancer HCT-8 cells, this study employed HE fruiting body powder as the raw material, with the extraction rate serving as the evaluation criterion. The optimal process model for SFE and the enrichment of the active compounds from HE fruiting body powder was determined through a single-factor test and the Box–Behnken response surface-optimization experiment. The versatility of the model was further verified by assessing the different varieties of HE. The potential biological activity of the supercritical HE extracts was assessed by investigating their anti-colon cancer activity against HCT-8 cells, as well as their antioxidant capacity, and antibacterial activity. Additionally, graded alcohol extraction was employed to separate the crude HE extracts and to confirm their anti-cancer efficacy. Concurrently, the extracts were analyzed using Fourier-transform infrared spectroscopy (FT-IR) and gas chromatography-mass spectrometry (GC-MS) techniques, with the three aforementioned indicators being utilized to compare the variations among the six HE varieties. This study represents the first documented report on the activity of HE against HCT-8 colon cancer cells, thus contributing to our current understanding in this field.

## 2. Results and Discussion

### 2.1. Optimization of the SFE Process of HE

#### 2.1.1. Screening Design

**Figure 1:**
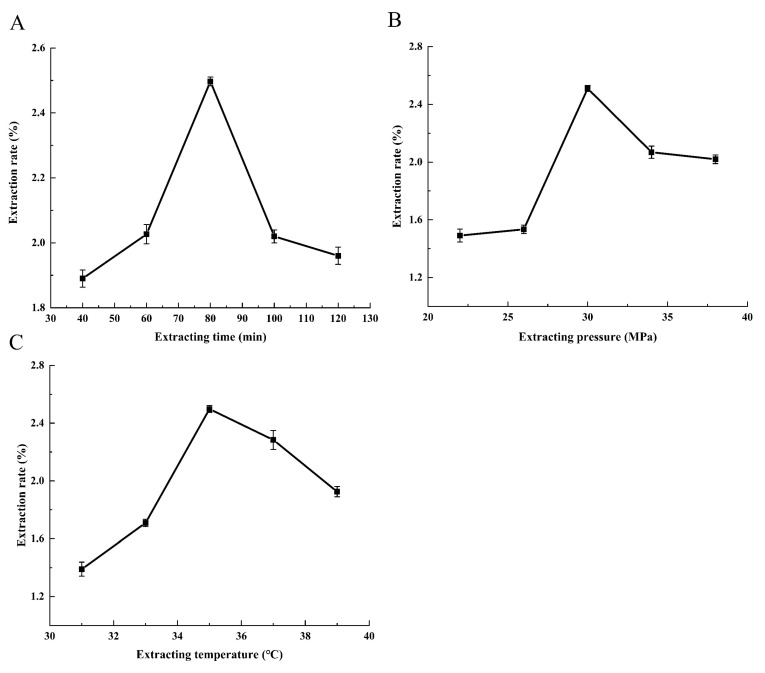
Single-factor test: the effect of the variables on the extraction rate. (**A**) Effect of extraction time on extraction rate, (**B**) effect of extraction pressure on extraction rate, and (**C**) effect of extraction temperature on extraction rate.

In accordance with our prior investigation, we identified three key factors namely extraction time, pressure, and temperature which exert a significant influence on the extraction rate. By referencing the pertinent literature [18,19,20,21], we established the approximate ranges for each of the factors’ values. Figure 1A–C presents the extraction rates that correspond to the various experimental conditions. Subsequently, we determined the optimal conditions for extraction, which involved the extraction times of 60, 80, and 100 min; pressures of 26, 30, and 34 MPa; and temperatures of 33, 35, and 37 °C. To refine these conditions further, we employed the Box–Behnken design (BBD). Our comprehensive analysis substantiates the statistical significance (*p* < 0.05) of all the factors under consideration.

#### 2.1.2. Optimization Design

This study employed a BBD to investigate the effects of the three significant factors (at three different levels): time (X_1_), pressure (X_2_), and temperature (X_3_). The center points for each variable were determined based on the results of a screening study (Table 1). Through a quadratic regression analysis, a statistically significant model was rated, thereby resulting in the optimized response surface equation: Y = 2.51 + 0.0204A + 0.1205B + 0.1160C − 0.0399AB + 0.0206AC − 0.1175BC − 0.2358A^2^ − 0.2451B^2^ − 0.1521C^2^. In the equation, Y represents the extraction rate, while A, B, and C represent the extraction time, pressure, and temperature, respectively. The analysis of variance (ANOVA) results (Table 2) revealed a strong relationship between the response and variables, with an adjusted *R*^2^ value of 86.55%. The model also demonstrated a high predictive ability for extraction rates, with a predicted *R*^2^ value of 94.12%. Based on the established mathematical model, an optimal condition analysis was conducted, thereby identifying the highest extraction rate at an extraction time of 80.8 min, a pressure of 30.7 MPa, and a temperature of 35.6 °C.

**Figure 2:**
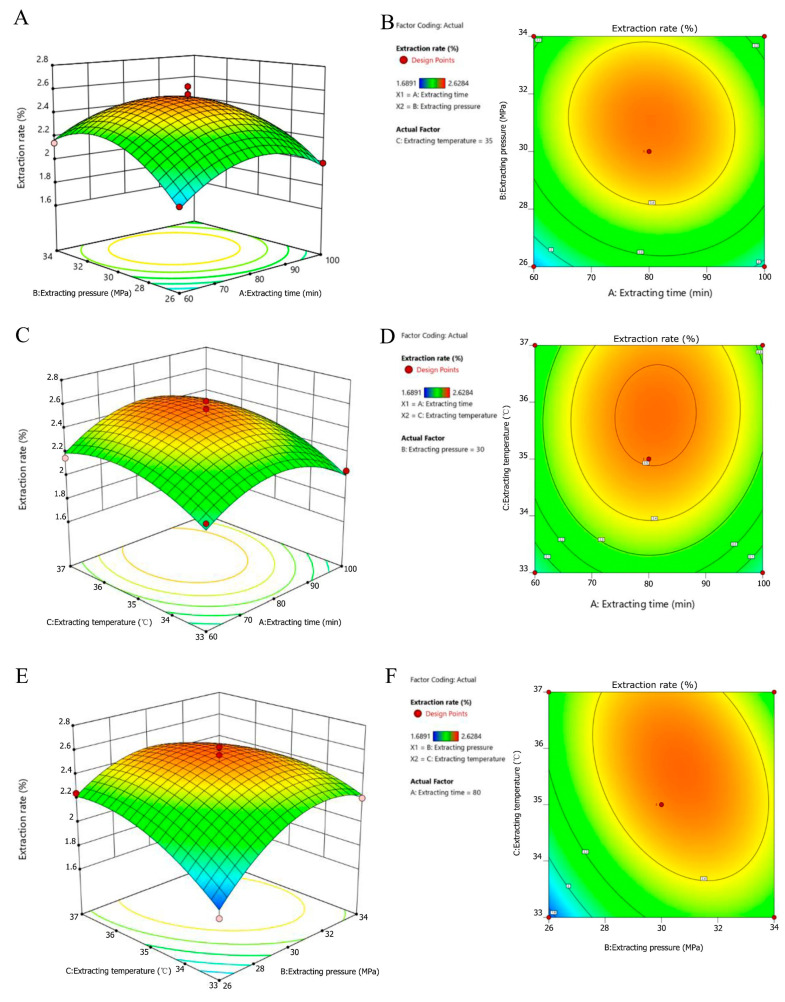
The response surface and contour plots illustrate the effects of the interactions among the extraction time, pressure, and temperature on the rate of extraction of a substance using supercritical CO_2_ extraction (SFE). The figure consists of six subplots: (**A**) Response surface plots of the effects of extraction time and extraction pressure on extraction rate. (**B**) Contour plot depicting the effect of extraction time and extraction pressure on extraction rate. (**C**) Response surface plots of the effects of extraction time and extraction temperature on extraction rate. (**D**) Contour plot illustrating the effect of extraction time and extraction temperature on extraction rate. (**E**) Response surface plots of the effects of extraction pressure and extraction temperature on extraction rate. (**F**) Contour plot showing the effect of extraction pressure and extraction temperature on extraction rate.

Figure 2 illustrates the estimated surface plots of the HE extraction. Decreasing the extraction time enhanced the extraction rate; this occurred because increasing the extraction temperature accelerates the thermal motion of solute molecules, thus improving their volatility and diffusion speed [22]. However, excessively high extraction temperatures reduce the density of supercritical CO_2_ fluid, leading to decreased solute solubility [23]. Prolonged extraction times can cause the volatilization of absolute ethanol, reducing the extraction rate due to a decreased entrained dose [24]. When the extraction temperature is low, increasing the extraction pressure raises the density of the supercritical CO_2_ fluid, enhancing its ability to dissolve polar substances and increasing the extraction rate [25]. However, extraction pressures below 20 MPa result in CO_2_ existing in a sub-supercritical state, which is unfavorable for normal SFE [26]. Considering the nature of supercritical extraction equipment, extraction pressures generally do not exceed 40 MPa [27]. Excessive extraction pressures, combined with higher extraction temperatures, excessively increase the density of SFE, thus diminishing the adsorption capacity of the extraction system and reducing the solubility of CO_2_ in the extract. This results in a lower extraction rate [28].

The predictive model was tested under optimized conditions, yielding, from the verification experiment (Table 3), a software-predicted value of 2.54% and an average value of 2.51%. The relative error of 2.68% was similar to the model-predicted value, confirming the accuracy and reliability of the optimized extraction process parameters that were obtained through the response surface method.

#### 2.1.3. Generality Analysis of the Model

**Figure 3:**
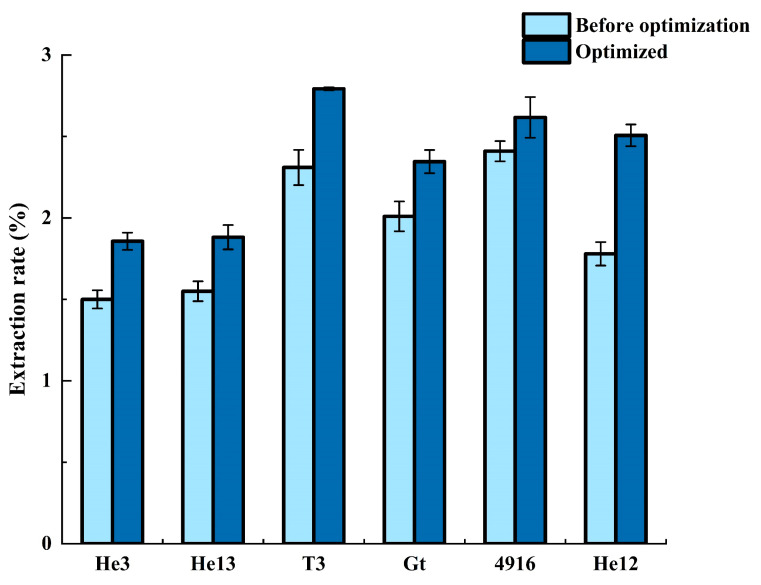
Comparison of the percentage extraction experiment before and after optimization of the six varieties of *Hericium erinaceus* (HE).

The response surface method was utilized to obtain the optimal conditions, which were then applied to the remaining five HE varieties in our laboratory. Table 4 presents the mean value and relative error of the extraction rate, along with a comparison of the extraction rate of the different HE varieties under the initial, unoptimized conditions (Figure 3). Prior to optimization, a single-sample variance analysis demonstrated a highly significant difference (*p* < 0.001) in the extraction rate of the different HE varieties when under identical conditions. Subsequently, a one-sample variance analysis was conducted on the extraction rate of the different HE varieties when under the optimized response surface conditions. The results from both analyses revealed a significant difference in the rate of the SFE among the various HE varieties. Interestingly, Figure 3 illustrates that there was no significant difference in the average growth rate of the extraction rate between the different varieties before and after optimization, thus confirming the model’s versatility for the SFE of various HE varieties.

### 2.2. Effects of HE Crude Extracts on the Inhibition of the HCT-8 Cell

**Figure 4:**
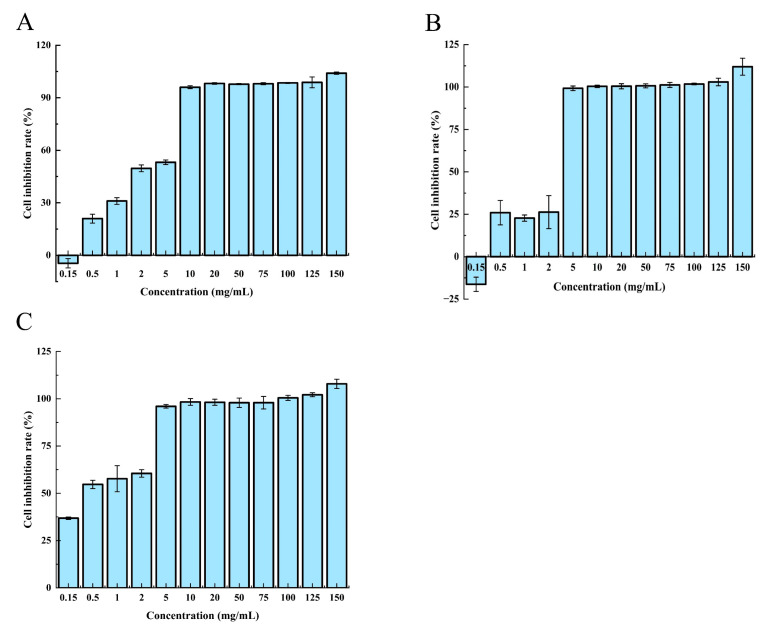
Anticancer activity diagram of the He12 supercritical crude extracts of HE at different culture times: (**A**) 24 h, (**B**) 48 h, and (**C**) 72 h.

To assess the potential anti-colorectal cancer activity of the crude extracts derived from HE, we conducted an MTT assay to evaluate their effect on the proliferation of HCT-8 cells. Figure 4 illustrates the administration of the various concentrations of HE crude extracts to HCT-8 cells for 24, 48, and 72 h. The results demonstrated a dose-dependent and time-dependent inhibitory effect of the crude extracts on HCT-8 cell growth, with a gradual increase in the inhibitory effect over time. Notably, at a concentration of 1.0 mg/mL, the cell growth inhibition rate reached 57.70%. These promising preliminary results prompted the selection of an appropriate concentration for further investigation into the anticancer activity of the crude extracts from six different varieties of HE.

**Figure 5:**
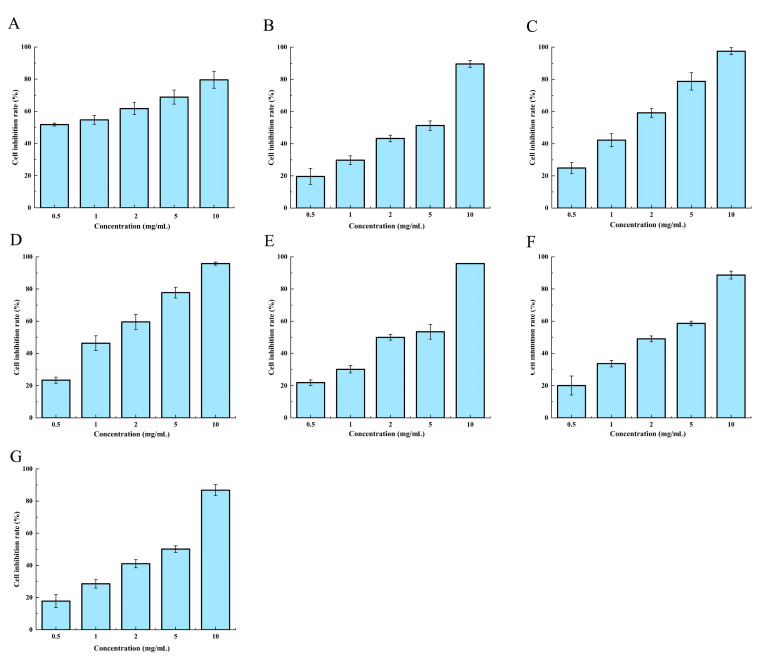
Anticancer activity diagram of the supercritical crude extracts of different varieties of HE (24 h). (**A**) Positive control 5-Fluorouracil, (**B**) 4916, (**C**) Gt, (**D**) He3, (**E**) He12, (**F**) He13, and (**G**) T3.

Accordingly, concentrations of 0.5, 1, 2, 5, and 10 mg/mL, combined with a culture time of 24 h, were chosen. Figure 5 presents the assessment of the inhibitory effect of these crude extracts on HCT-8 cells. The resulting IC_50_ values were determined as follows: the positive control 5-Fu had an IC_50_ of 0.528 mg/mL, He13 had an IC_50_ of 2.224 mg/mL, He12 had an IC_50_ of 2.255 mg/mL, 4916 had an IC_50_ of 2.709 mg/mL, T3 had an IC_50_ of 2.971 mg/mL, Gutian (Gt) had an IC_50_ of 1.356 mg/mL, and He3 had an IC_50_ of 1.315 mg/mL. Notably, at a concentration of 2 mg/mL, the cell inhibition rates were 61.75% for 5-Fu, 59.54% for He3, and 59.17% for Gt. These findings confirm the existence of a certain inhibitory effect that is caused by the HE crude extracts on HCT-8 cells.

### 2.3. Antioxidant and Antibacterial Activities of the Crude Extracts from HE by Using SFE

#### 2.3.1. Antiradical Activities

**Figure 6:**
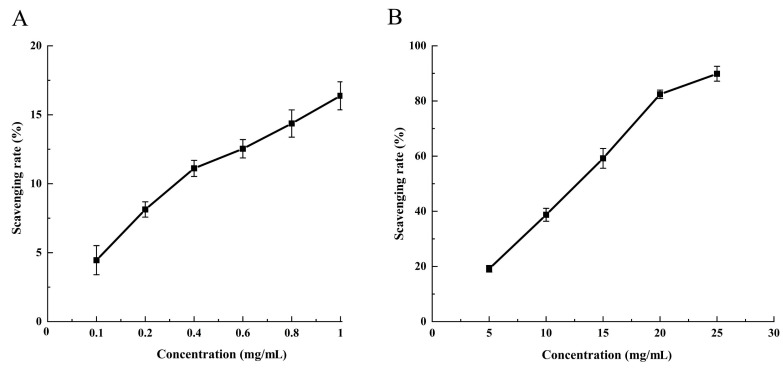
Antioxidant activity of the supercritical crude extracts of HE. (**A**) Low sample concentration, (**B**) high sample concentration.

The antiradical activities of HE extracts obtained through SFE were assessed using the 1,1-diphenyl-2-picrylhydrazyl (DPPH) radical scavenging assay, which is a reliable and reproducible method that is commonly used to evaluate the in vitro antiradical activities of pure compounds and extracts [29]. DPPH, a stable free radical in ethanol, forms a violet solution that turns colorless when it reacts with antioxidant compounds, thereby indicating reduction [30]. Figure 6 presents the DPPH radical scavenging activities of the HE crude extracts. These extracts demonstrated dose-dependent scavenging activities against DPPH radicals at both low and high concentrations. However, the results revealed a limited antioxidant activity at an extract concentration of 1 mg/mL, with approximately 16.86% DPPH radical scavenging activity. This outcome can be attributed to the non-polar nature of the solvent [31], resulting in the extraction of primarily non-polar components with low antioxidant activity.

#### 2.3.2. Antibacterial Activities

The inhibition zone method, also referred to as the diffusion method, involves the diffusion of a test substance within the agar plate so as to impede the growth of nearby bacteria, thus resulting in the formation of a transparent circle. The size of the inhibition zone is used to determine the antibacterial effectiveness of the test substance [32,33]. In this study, we employed the inhibition zone method to evaluate the bacterial inhibition capabilities of the HE extracts.

The He12 supercritical extracts were tested against *Escherichia coli*, *Staphylococcus aureus*, *Bacillus subtilis*, and *Sphaeromyces cerevisiae*. However, no antimicrobial activity was observed in the He12 supercritical crude extracts against *B*. *subtilis* and *S*. *cerevisiae* as evidenced by the absence of an inhibition zone or a zone smaller than 9 mm. Table 5 illustrates the results of the agar diffusion essays for the extracts tested against the studied microorganisms in terms of the size of the inhibition zone (mm). The results indicate that the crude extract concentration of 25 mg/mL exhibited an inhibitory effect of 18 mm on *E*. *coli*. Conversely, at concentrations of 1 mg/mL and 10 mg/mL, no inhibition zone or an inhibition zone smaller than 9 mm was observed. Similarly, at concentrations of 1 mg/mL and 25 mg/mL, no inhibition zone or an inhibition zone smaller than 9 mm was detected for *S*. *aureus*, except for a concentration of 10 mg/mL, which displayed an inhibitory effect of 17 mm.

The antimicrobial analysis revealed that the supercritical crude extracts of HE possess a certain level of antibacterial activity against *E*. *coli* (G^−^) and *S*. *aureus* (G^+^), but no antibacterial activity against *B*. *subtilis* (G^+^). The antibacterial activities of the crude extracts of HE obtained through SFE vary depending on the specific microorganism being targeted.

### 2.4. Effects of HE Fractional Alcohol Extracts on the Inhibition of HCT-8 Cells

**Figure 7:**
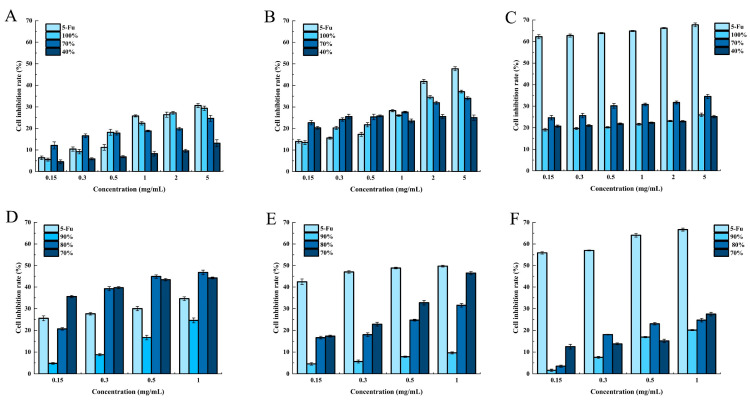
The anti-cancer activity of the crude graded ethanol extracts of HE He12 under different culture times: (**A**) 24 h, (**B**) 48 h, and (**C**) 72 h. The anti-cancer activity figure of the secondary graded ethanol extracts of HE He12 under different culture times: (**D**) 24 h, (**E**) 48 h, and (**F**) 72 h.

Coarse-graded alcohol extraction was utilized to fractionate the supercritical crude extracts of HE into four fractions: 100% alcohol precipitate, 70% alcohol precipitate, 40% alcohol precipitate, and 40% alcohol supernatant. The anticancer activity of the fractionated alcoholic extracts derived from HE He12 crude was assessed, as shown in Figure 7A–C. Varying concentrations of the isolated and fractionated alcoholic extracts were applied to HCT-8 cells for 24, 48, and 72 h. The growth inhibitory effect of the HCT-8 cells exhibited a dose-dependent and time-dependent relationship, except for the 40% alcohol supernatant, which exhibited no anticancer activity and was thus omitted from the figure. These findings suggest that the primary components with anticancer activity in the crude extracts of HE are concentrated in the 70% alcohol precipitate and higher fractions.

Based on the results obtained from the coarse fractional alcohol extraction, a secondary fractional alcohol extraction was conducted to partition the supercritical crude extracts of HE into three fractions: 90% alcohol precipitate, 80% alcohol precipitate, and 70% alcohol precipitate. The anticancer activity of the HE He12 secondary fractionation ethanol extracts was evaluated and is depicted in Figure 7D–F. Various concentrations of the isolated and secondary fractionated alcohol extracts were applied to the HCT-8 cells for 24, 48, and 72 h. The results demonstrated a dose-dependent inhibitory effect that was caused by the crude extracts of HE on the growth of HCT-8 cells. Specifically, the 90% alcohol precipitate exhibited lower anticancer activity when compared to the 80% and 70% alcohol precipitates. Further comparison revealed similar anticancer activity between the 80% and 70% alcohol precipitates. As a result, the 70% alcohol precipitate was chosen for subsequent experiments.

**Figure 8:**
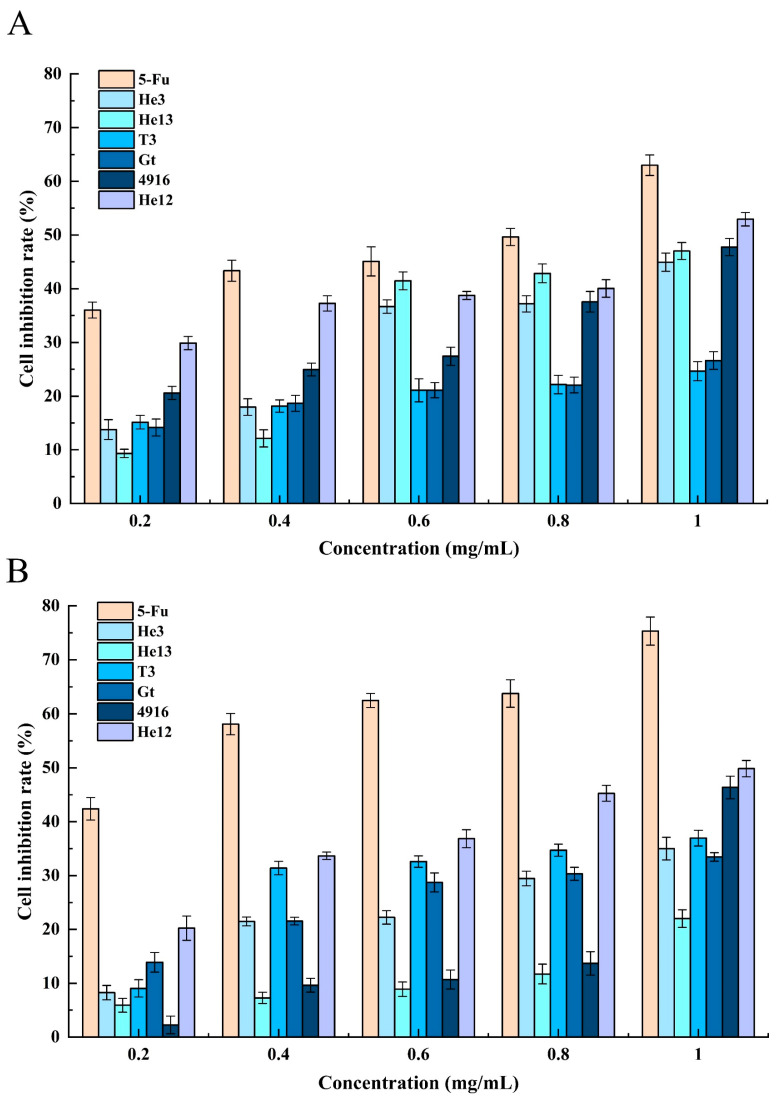
Anticancer activity of the graded ethanol extracts from the different varieties of HE. (**A**) Anticancer activity of the fractionated ethanol extracts of the six HE varieties for 24 h, (**B**) anticancer activity of the fractionated ethanol extracts of the six HE varieties for 48 h.

In accordance with the findings obtained from graded alcohol extraction, the growth inhibitory effect of the 70% alcohol precipitates that were induced by the six different varieties of HE on HCT-8 cells was examined, as presented in Figure 8. The HCT-8 cells were individually exposed to various 70% alcohol precipitate concentrations and varieties of the HE cells for 24 and 48 h. The results demonstrated a dose-dependent inhibition of HCT-8 cell growth by the 70% alcohol precipitates from the six HE varieties. Notably, when the concentration of the 70% alcohol precipitate from the HE He12 variety was 1.0 mg/mL, the rate of the cell growth inhibition reached 51.56%. Under the same concentration conditions, the positive control 5-Fu exhibited a cell growth inhibition rate of 60.98%. These findings highlight the successful separation of the active components in the crude extracts of HE through fractional alcohol extraction.

### 2.5. Functional Groups in HE Crude Extracts

The functional groups present in the crude extracts of HE were determined using FT-IR, a widely employed technique for evaluating substance quality and for conducting chemical analysis [5].

**Figure 9:**
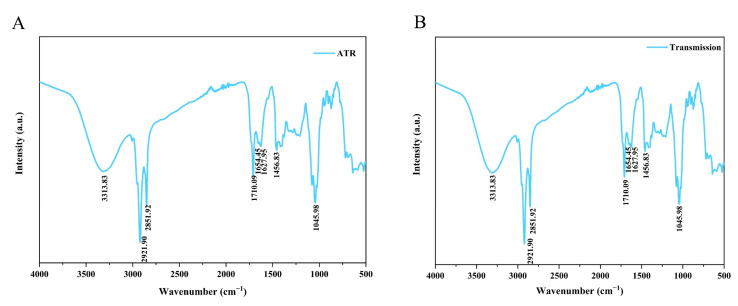
Fourier-transform infrared spectroscopy (FT-IR) spectra of the supercritical crude extracts of HE. (**A**) ATR and (**B**) transmission.

The main functional groups in the HE crude extracts are identified in Figure 9A (ATR) and Figure 9B (transmission). Two detection methods (ATR and transmission) were employed within the wavenumber range of 4000 cm^−1^ to 500 cm^−1^. Figure 9A,B clearly depicts broad peaks at 3313.83 cm^−1^, indicating the presence of intermolecular hydroxyl (-OH) groups undergoing tensile vibrations. A sharp peak at 2921.90 cm^−1^ was identified as dimer acid, while a medium sharp absorption band at 2851.92 cm^−1^ indicated the presence of aldehydes, specifically carbonyl groups (C=O). The sharp absorption bands at 1710.09 cm^−1^ were assigned to ketones, which could encompass six-membered ring ketones, seven-membered ring ketones, or larger ring ketones. Two weak peaks, observed between 1600 cm^–1^ and 1700 cm^–1^ (specifically at 1627.95 cm^−1^ and 1654.45 cm^−1^), were identified as nitrites. Furthermore, a weak absorption band at 1456.83 cm^−1^ was identified as cis amides. Another sharp absorption band at 1045.98 cm^−1^ in the HE extracts indicated the stretching vibration of the amine groups (C=N). The results derived from FT-IR established a theoretical basis for the subsequent experiments involving component analysis.

### 2.6. Component Analysis of the Extracts in Relation to Bioactivities

**Figure 10:**
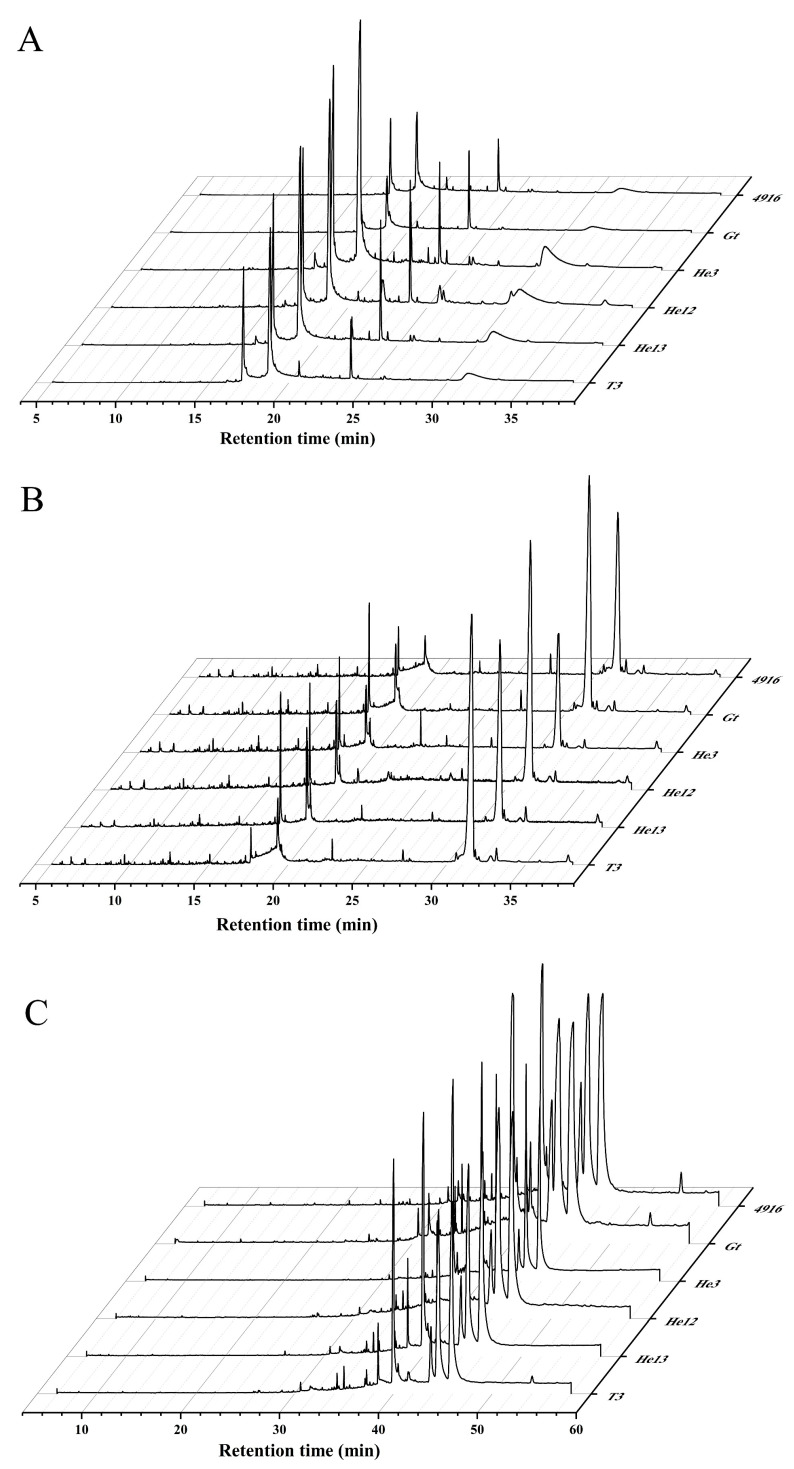
Gas chromatography-mass spectrometry (GC-MS) diagram of supercritical crude extract and ethanol precipitate of HE. (**A**,**C**) are detected using different instruments and methods. (**A**) Ion chromatograms of the supercritical crude extracts of the different HE varieties. (**B**) Ion chromatograms of the graded ethanol precipitate of different HE varieties. (**C**) Ion chromatograms of the supercritical crude extracts of the different HE varieties.

**Figure A1:**
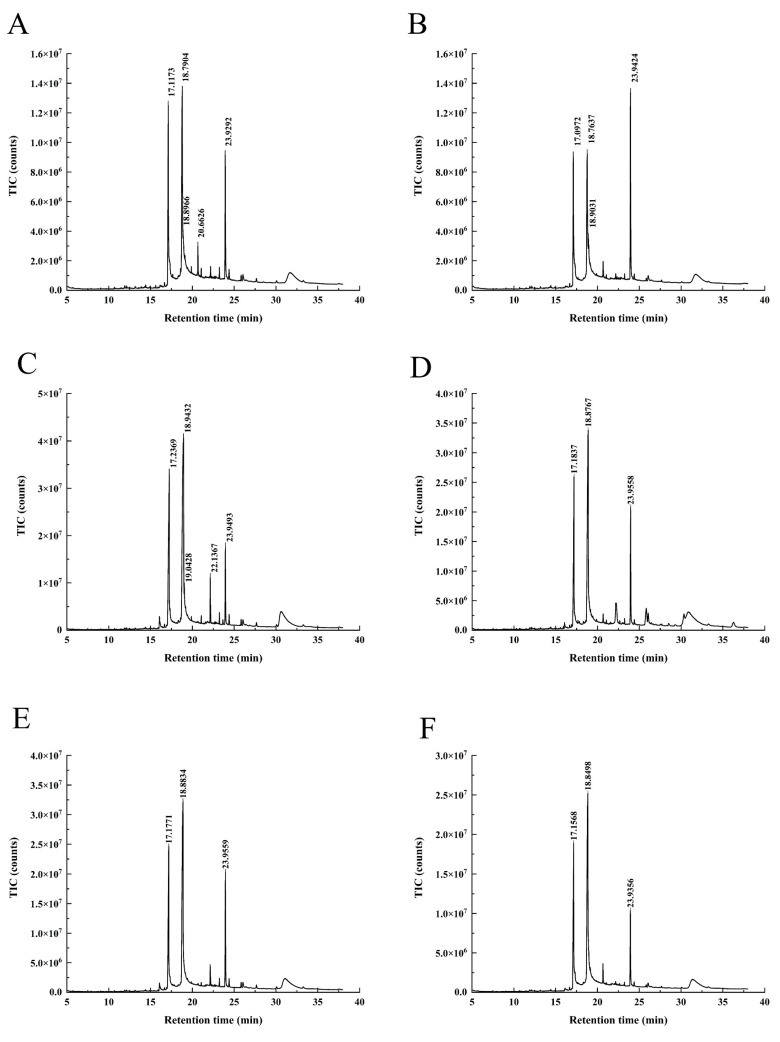
GC-MS spectra of the supercritical crude extracts of the different HE varieties. (**A**) 4916, (**B**) Gt, (**C**) He3, (**D**) He12, (**E**) He13, and (**F**) T3.

**Figure A2:**
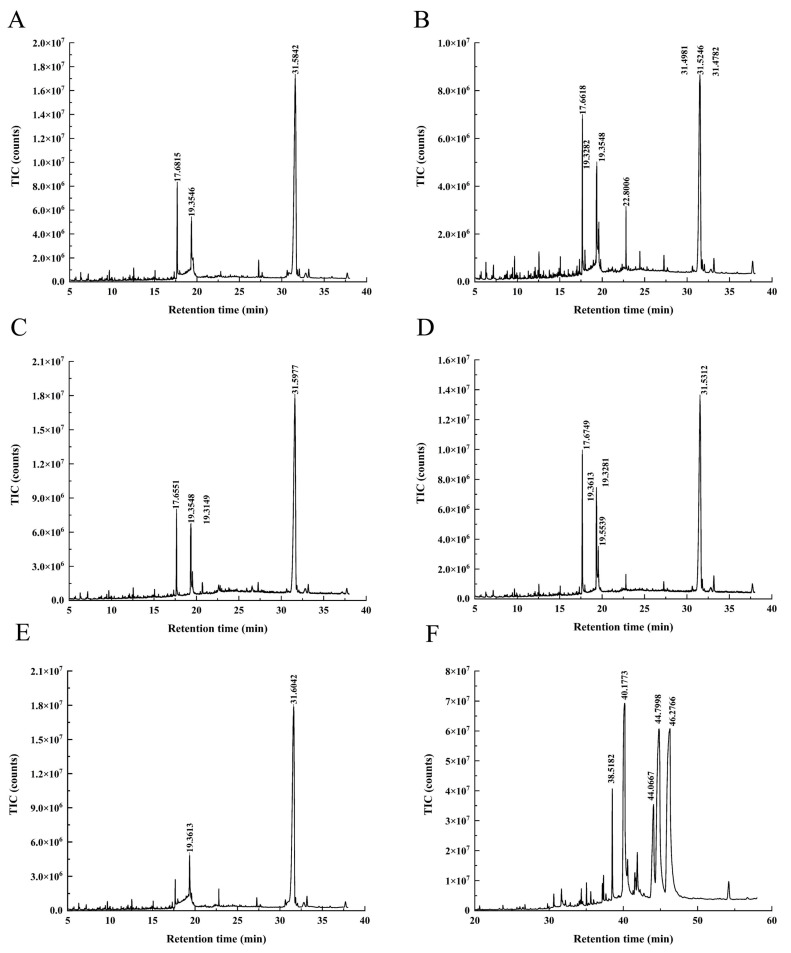
GC-MS spectra of the 70 % alcohol precipitation of the different HE varieties. (**A**) 4916, (**B**) Gt, (**C**) He3, (**D**) He12, (**E**) He13, and (**F**) T3.

**Figure A3:**
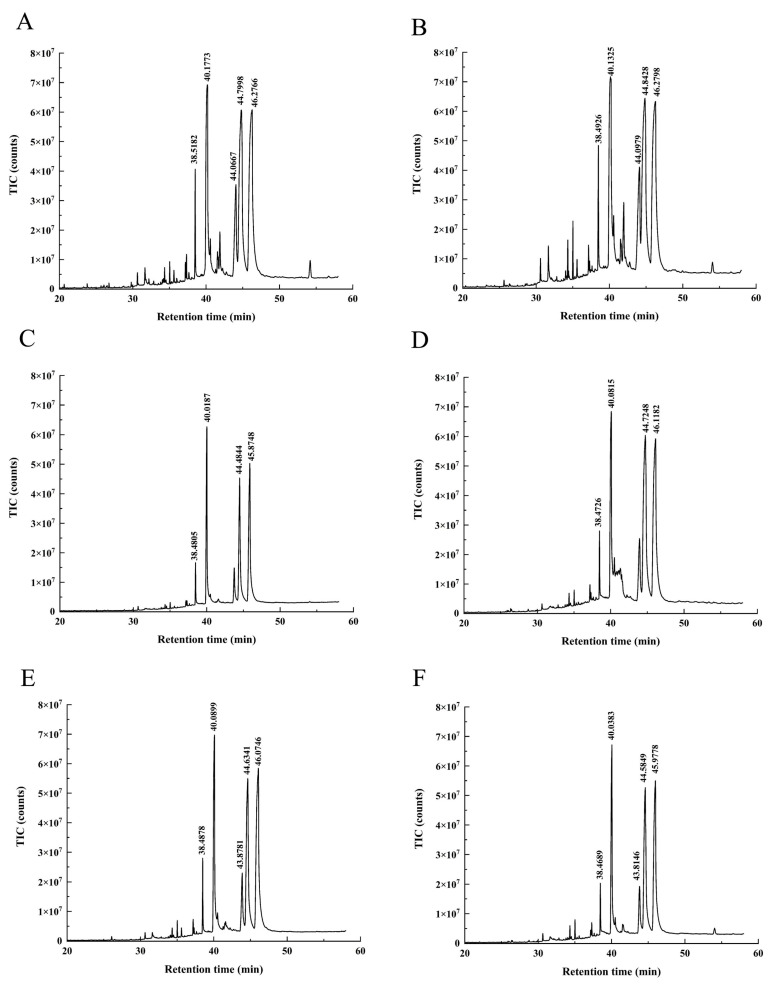
GC-MS spectra of the supercritical crude extracts of the different HE varieties (Shimazu). (**A**) 4916, (**B**) Gt, (**C**) He3, (**D**) He12, (**E**) He13, and (**F**) T3.

The peaks derived from the supercritical crude extracts of HE were subjected to GC-MS analysis (Agilent) by using the standard spectrum library to determine the composition of each peak. The chemical constituents, which were identified from a single variety of HE, are shown in Appendix A Figure A1, Figure A2 and Figure A3. The GC-MS spectrum of the crude supercritical extracts of HE is presented in Figure 10A. The main components identified in the 4916 crude extracts were palmitic acid, oleic acid, 9-octadecenamide, erucamide, dehydro-ergosterol, and ergosterol. In Gt, the primary components were palmitic acid, octadecadienoic acid, oleic acid, erucamide, isochoric acid ethyl ester, dehydro-ergosterol, and ergosterol. He3 contained pentadecanoic acid, palmitic acid, erucamide, and ergosterol as its main components, while He12 comprised pentadecanoic acid, palmitic acid, isochoric acid ethyl ester, erucamide, dehydro-ergosterol, and ergosterol. The main components of He13 were pentadecanoic acid, palmitic acid, erucamide, dehydro-ergosterol, and ergosterol, while T3 consisted of palmitic acid, octadecadienoic acid, 9-octadecenamide, erucamide, dehydro-ergosterol and ergosterol. Among the identified components, stearic acid esters were the most common, with stearic acid methyl esters being the predominant ones. Notably, this study detected erucamide in HE for the first time. Erucamide is commonly used as a slip agent and as an anti-adhesive agent in plastics; thus, this could indicate potential environmental pollution during the growth of the HE. Palmitic acid and oleic acid, which exhibited the highest relative content, are crucial components in the growth process of HE. As an octadecyl compound, oleic acid is widely distributed in organisms and possesses significant physiological activities.

To investigate further, a secondary detection was carried out with different methods and instruments (Shimadzu, Kyoto, Japan) on the supercritical extracts of the six HE varieties. Notably, significant variations were observed in the components of the supercritical crude extracts across the different HE varieties (see Supplementary Materials, Tables S1–S6). Specifically, 4916 exhibited 47 components, Gt had 52 components, He3 contained 31 components, He12 contained 38 components, He13 contained 42 components, and T3 contained 38 components. Based on the GC-MS analysis of the chemical structure, the components were broadly categorized into three groups: hydrocarbons, oxygen-containing compounds, and nitrogen-containing compounds. The GC-MS spectrum of the supercritical crude extract of HE is shown in Figure 10C. Analysis of the peak areas revealed that the five components with the highest concentrations of the six HE varieties were 9,12-octadecadienoic acid, oleic acid, stearic acid, palmitic acid, and pentadecanoic acid. Previous studies on the volatile oil components of HE have predominantly focused on fatty acids and esters, such as palmitic acid, stearic acid, oleic acid, etc.

The GC-MS spectrogram in Figure 10B illustrates the HE isolates obtained through fractional alcohol extraction. The primary constituents of the 4916 isolates were caproic acid, trimethyl tetradecane, palmitic acid, dehydro-ergosterol, neo-ergosterol, and ergosterol. Similarly, Gt contains caproic acid, phenethyl-benzylamine, palmitic acid, octadecadienoic acid, dehydro-ergosterol, spiro sterane, ergosterol, neo-ergosterol, ergo-sterane, and anthracene tetra enol. He3 consists of caproic acid, palmitic acid, oleic acid, spiro sterane, dehydro-ergosterol, ergosterol, and neo-ergosterol. In addition, He12 includes caproic acid, stearic acid, palmitic acid, dehydro-ergosterol, ergosterol, ergo sterane, and γ-ergosterol, while He13 contains benzene, hexanoic acid, trimethyl tetradecane, palmitic acid, spiro sterane, dehydro-ergosterol, ergosterol, ergo-sterane, γ-ergosterol, and anthracene tetra enol. T3 consists of hexanoic acid, palmitic acid, octadecadienoic acid, spiro sterane, dehydro-ergosterol, ergosterol, neo-ergosterol, anthracene tetra enol, γ-ergosterol, and ergo sterane. Upon comparing the GC-MS spectrum of the crude extracts, erucamide was found to be absent in the HE isolates. However, various sterols and sterol derivatives were detected, including ergosterol, dehydro-ergosterol, neo-ergot sterols, γ-ergosterol, ergo-steranes, spiro sterane, and anthracene tetra enol. Due to the diverse chemical properties of these identified compounds, it remains unclear which specific compounds or combinations contribute to the inhibition of HCT-8 cell proliferation. Hence, further validation studies are necessary to determine the therapeutic potential of the active ingredients present in the supercritical extracts of HE.

## 3. Materials and Methods

### 3.1. Reagents and Chemicals

The following six varieties of HE were cultivated in our laboratory: 4916, Gt, He3, He12, He13, and T3. Absolute ethanol was obtained from Fangzheng Reagent Factory (Tianjin, China). The human colonic carcinoma cell line HCT-8 was acquired from the research group led by Liu Xingzhong at Nankai University (Tianjin, China). Dulbecco’s modified Eagle’s medium (DMEM), fetal bovine serum (FBS), dimethyl sulfoxide (DMSO), and RPMI 1640 were sourced from GIBCO (Grand Island, NY, USA). The MTT reagent (3-(4,5-dimethylthiazol-2-yl)-2,5-diphenyltetrazolium bromide) was purchased from Biyuntian Biotechnology Co., Ltd. (Shanghai, China). The DPPH free radical scavenging ability kit was obtained from the Jiancheng Bioengineering Institute (Nanjing, China). All of the chemicals and solvents used were of high purity and of an analytical grade.

### 3.2. Sample Preparations

The fruiting body of HE was harvested and oven-dried at a constant temperature of 50 °C until the moisture content stabilized at 10% (*w*/*w*). Subsequently, the dried fruiting body of HE was finely ground using a grinder and was then sifted through a 0.85 mm mesh sieve to obtain a powdered form. For specific details about the characteristics of the materials, please refer to Table 6.

### 3.3. Supercritical Fluid Extraction

**Figure 11:**
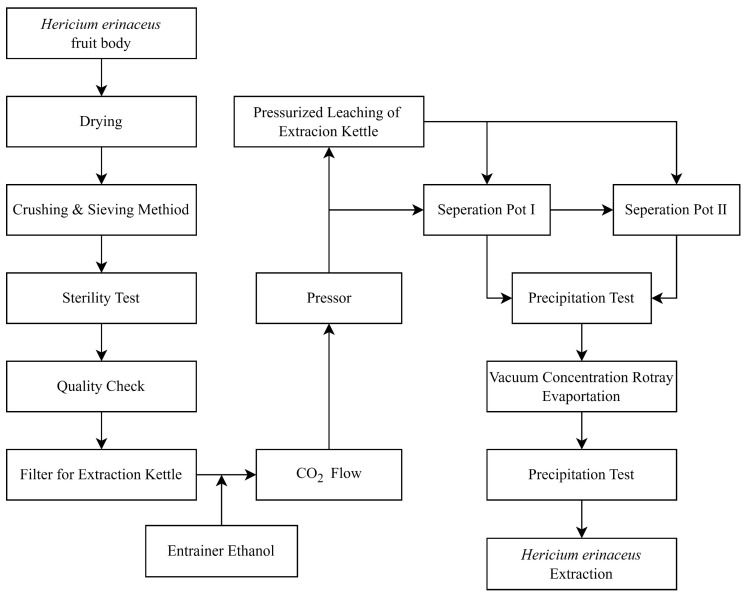
Supercritical CO_2_ extraction flow chart of the HE fruiting body.

**Figure 12:**
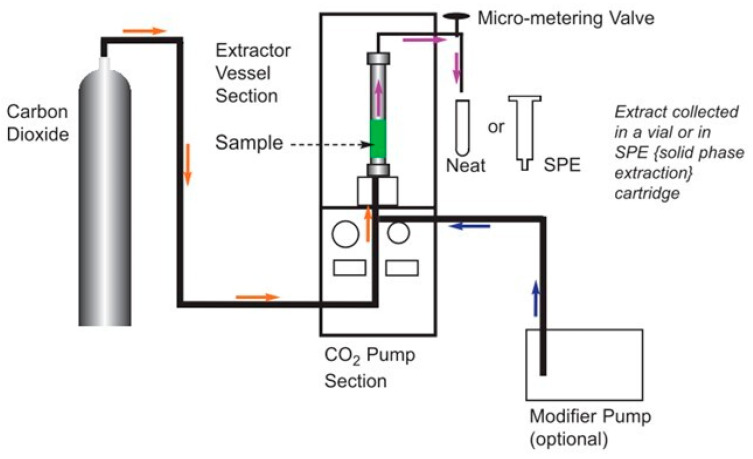
Structure diagram of the SFE instrument, the orange arrow indicates that the CO_2_ gas flows from the cylinder into the extraction kettle. The purple arrow indicates that after the mixed extraction of the sample and CO_2_ is completed, the extraction sample flows into the collection bottle and the gas is put into the atmosphere. The blue arrow represents the flow of compressed air driven by a compressed air pump, which assists in pressurization. (Source: Applied Separations, Inc., Allentown, PA, USA).

The process of the SFE of the HE is depicted in Figure 11, whereby the step-by-step procedure employed with the Spe-ed SFE Basic extractor (Applied Separations, Allentown, PA, USA) is demonstrated. For a detailed representation of the equipment utilized, including its schematic diagram, please refer to Figure 12.

#### 3.3.1. Screening Design

During each extraction, approximately 6 g of HE fruiting body powder was loaded into an extraction thimble and placed within the extraction chamber. The modifier used was ethanol absolute, which was used in accordance with the laboratory’s previous research foundation and was utilized at a volume of 12 mL. The experimental design incorporated three variable factors: extraction time (A: 40, 60, 80, 100, and 120 min); pressure (B: 22, 26, 30, 34, and 38 MPa); and temperature (C: 31, 33, 35, 37, and 39 °C). Subsequently, the resulting extract was collected in a flask and stored at −20 °C until required for further analysis of the extract rate and bioactive components. The percentage extract rate was determined by subjecting the extract to vacuum rotary evaporation at 45 °C until a constant weight of the dried extract was attained.

#### 3.3.2. Optimization Design

BBD was implemented to arrange the experiments that were aimed at optimizing the response surface for the extraction of bioactive compounds from the HE fruitbodies using SFE [34]. This study investigated the impact of SFE extraction time, pressure, and temperature concentration on the recovery process. Based on the findings in Section 3.3.1, the extraction times of 60, 80, and 100 min were tested, along with the pressures of 26, 30, and 34 MPa, and the temperatures of 33, 35, and 37 °C. The response surface method was employed for factor analysis and was conducted with a horizontal design, as illustrated in Table 7. The levels for each process variable were determined through a series of preliminary trials. Additionally, the response surface analysis was conducted on the data obtained from the array design to develop a predictive model and to determine the optimal conditions for SFE, specifically in relation to the extraction rate from the HE fruit bodies.

### 3.4. Determination of Antiradical Activity

The assessment of the free radical scavenging rate involved the utilization of DPPH with V_C_ as the reference standard. To initiate the analysis, the HE extracts were dissolved in ethanol and combined with 600 µL of a DPPH radical working solution, which was obtained from the Nanjing Jiancheng Bioengineering Institute, Nanjing, China. The resulting mixture was incubated for a duration of 30 min at a temperature of 25 °C, under conditions of light exclusion. Subsequently, the absorbance measurements were performed at a wavelength of 517 nm with a UV-visible spectrophotometer. The percentage inhibition was calculated by employing the following formula: [1 − (A_2_ − A_1_)/A_0_] × 100%. In this formula, A_0_ represents the absorbance of the blank, A_1_ corresponds to the absorbance of the control, and A_2_ represents the absorbance of the sample.

### 3.5. Antimicrobial Activity

#### 3.5.1. Microorganism Preparation

The HE extracts obtained via SFE were evaluated for their antimicrobial activity against bacterial strains, including *E*. *coli*, *S*. *aureus*, *B*. *subtilis*, and *S*. *cerevisiae*. These bacterial strains were sourced from the microbiology laboratory at Nankai University. Incubation of the cultures occurred at 37 °C, except for the yeast cultures (which were incubated at 25 °C). Additionally, all bacterial cultures were maintained under aerobic conditions.

#### 3.5.2. Antibacterial Test

In accordance with the medium configuration formula, the sterilized medium was dispensed into Petri dishes, with an approximate volume of 20 mL per plate. The plates were subsequently inverted and placed in a 37 °C incubator for a two-day cultivation period. In the event that no colonies meeting the required standards were observed, the next experiment would be carried out. A specific quantity of the HE extracts was carefully weighed, and absolute ethanol was employed as the solvent. The resulting mixture of the bacteria and medium was then poured onto a culture medium plate, which was positioned on a sterilized Oxford cup that already contained the culture medium. After the culture medium solidified, a dropper was employed to draw up 0.3 mL of the prepared solution. Subsequently, the Oxford cup, now containing 0.3 mL of the solution at various concentrations, was placed in a 37 °C incubator to undergo a proper time cultivation process. The diameter of the resulting bacteriostatic circle was measured and accurately recorded with an electronic vernier caliper.

### 3.6. Cell Culture and Viability Assay

#### 3.6.1. Cell Culture

The HCT-8 cells were cultured in an RPMI 1640 medium, which was supplemented with 10% fetal bovine serum (FBS), glutamine, and antibiotics (100 mL units of penicillin and 100 µg/mL of streptomycin). The cells were incubated at 37 °C in a 5% CO_2_ atmosphere.

#### 3.6.2. Cell Viability

Following a pre-incubation period of 12 h, the HCT-8 cells (4 × 10^3^ cells/mL) were subjected to treatment with the HE extracts or with the isolated compounds. They were then subsequently incubated for 24, 48, and 72 h. The cytotoxic effects of the HE extracts and the isolated compounds were evaluated using a conventional MTT assay. To terminate the cell cultures, a 20 µL solution of MTT (5 mg/mL in phosphate-buffered saline, pH 7.4) was added, and the cells were cultured for an additional 5 h. The incubation process was halted by introducing 150 µL of DMSO into each well to dissolve the resulting formazan. Subsequently, the absorbance at 490 nm (OD_490_) was measured using an enzyme calibration.

Cytotoxicity (%) = [1 − (absorbance of extract treated/absorbance of PBS treated cells)] × 100%.

### 3.7. Graded Alcohol Extraction

#### 3.7.1. Crude Graded Alcohol Extraction

**Figure 13:**
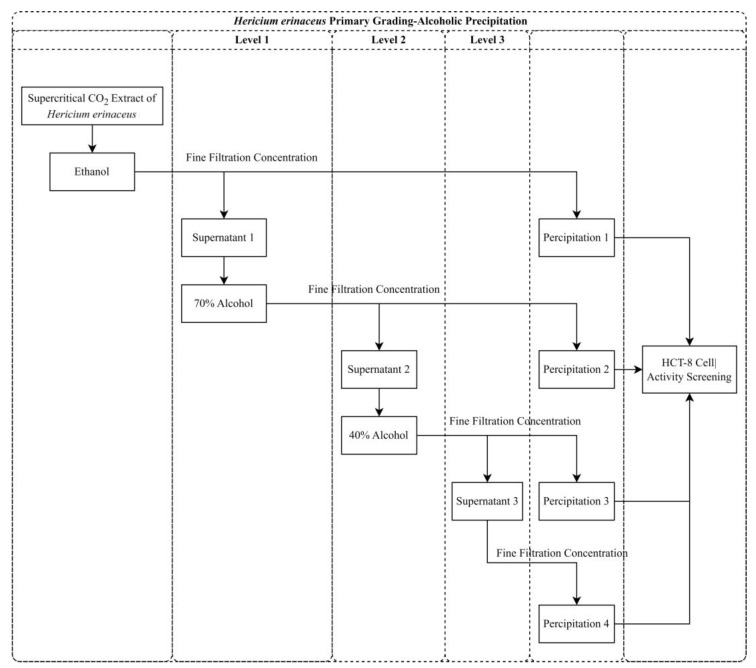
Flow chart of the crude grading alcohol extraction of the HE extracts.

Initially, the crude extracts of HE were dissolved in absolute ethanol to prepare a solution with a concentration of 0.1 mg/mL, and this was conducted utilizing ultrasound and stirring when necessary. Subsequently, the appropriate volumes of deionized water were added to the ethanol extracts so as to achieve the final ethanol concentrations of 70% and 40% [35]. To ensure purity and minimize evaporation, the solutions were carefully sealed with parafilm. Following this, the solutions were left undisturbed at 4 °C for a duration of 12 h. The separation of the supernatant and precipitate was achieved through centrifugation at 12,000 r/min for 15 min. The supernatant, collected for further experimentation, underwent rinsing three times before undergoing concentration to remove the ethanol; subsequently, it was dried into a powder. The remaining 40% of the supernatant was subjected to vacuum concentration and a subsequent drying into powder for further experiments. The process of the crude graded alcohol extraction is visually represented in Figure 13.

#### 3.7.2. Secondary Graded Alcohol Extraction

**Figure 14:**
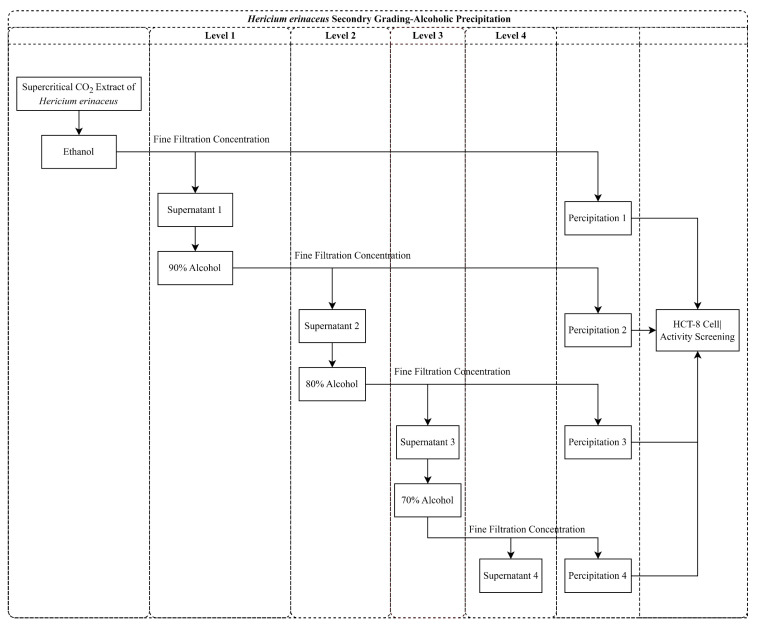
Flow chart of the secondary grading alcohol extraction of the HE extracts.

According to the findings of the initial crude graded alcohol extraction, we proceeded to perform the subsequent step of secondary graded alcohol extraction. Initially, the crude HE extracts were dissolved in absolute ethanol, resulting in a solution with a concentration of 0.1 mg/mL. Ultrasound and stirring techniques were employed as necessary during the process. To obtain ethanol concentrations of 90%, 80%, and 70% in the extracts, suitable amounts of deionized water were added. To prevent contamination and to minimize evaporation, the solutions were carefully sealed with parafilm [36]. Subsequently, the solutions were allowed to stand undisturbed for 12 h at a temperature of 4 °C. The resulting mixture was then subjected to centrifugation at 12,000 r/min for 15 min, thus separating the supernatant and precipitate. The supernatant was collected for further experiments, while the precipitate underwent three rounds of rinsing, followed by concentration to remove the ethanol; finally, it was dried so as to obtain a powdered form. Figure 14 provides a visual representation of the detailed process of the secondary graded alcohol extraction.

### 3.8. Cell Culture and Viability Assay

Conducted in the same manner as detailed in Section 3.5.

### 3.9. Fourier-Transform Infrared Spectroscopy (FT-IR)

The FT-IR spectroscopy spectra of the HE extracts were acquired using a Thermo Fisher Scientific (Waltham, MA, USA) Nicolet iS50 spectrophotometer. The analysis encompassed a range of 400 to 4000 cm^−1^, with a resolution of 4 cm^−1^. To ensure uniformity and reliability, the sample underwent a preparation step where it was mixed with 400 mg of KBr and then subsequently subjected to grinding and tableting processes.

### 3.10. Component Analysis Using GC-MS

Each hexane extract underwent an analysis that was conducted using a gas chromatograph, specifically the Agilent 19091S-433 model (Agilent Technologies, Inc., Santa Clara, CA, USA), which was equipped with an Hp-5MS column measuring 29.9 m × 0.25 mm × 0.25 µm. The temperature was programmed to begin at 70 °C for 2 min, followed by a linear increase of 10 °C per minute until reaching 280 °C (which was maintained for 15 min). Helium gas was utilized as the carrier with a flow rate of 1 mg/mL. Both the injector and detector temperatures were set at 250 °C. The injection volume was 1 µL. Identification of the components was performed by analyzing their retention times in the mass spectra and by comparing them with the mass spectra in a commercially available library.

In order to comprehensively study the active ingredients in HE, a secondary detection was carried out using different methods and instruments (Shimadzu) on the supercritical extracts of the six HE varieties. Each extract from the HE underwent an analysis that was conducted using a gas chromatograph, specifically the GCMS-QP 2010 model (Shimadzu Corporation, Japan), which featured an Hp-wax column with dimensions of 30 m × 0.25 mm × 0.25 µm. The temperature was programmed as follows: initially set at 50 °C for 1 min, then increased at a rate of 5 °C per minute until reaching 180 °C (which was maintained for 1 min), and then finally further increasing at a rate of 7 °C per minute until reaching 250 °C (which was maintained for 20 min). Helium gas was employed as the carrier, flowing at a rate of 1 mg/mL. Both the injector and detector temperatures were maintained at 250 °C. A volume of 1 µL was injected. Components were identified by comparing their retention times in the mass spectra with those present in a commercial library.

### 3.11. Statistical Analyses

The data analysis was performed using SPSS Statistics 26.0. A one-way ANOVA was utilized to compare the means across various groups, followed by the LSD-*t* test. To determine the correlation of the data and to pinpoint the optimal condition, Design Expert 12 software was employed to generate response surface equations, response surface plots, and main effect plots. Graphs were created using Origin 2018 (Version number: b9.5.1.195). Statistical significance was established at *p* < 0.05.

## 4. Conclusions

In this study, we utilized SFE technology to isolate the bioactive compounds from the fruiting body of HE. The extraction process was optimized, revealing that the highest extraction rate was achieved with an extraction time of 80 min, a pressure of 30 MPa, and a temperature of 35 °C. Notably, we made a novel discovery that the SFE of HE displays remarkable inhibitory activity against colon cancer HCT-8 cells, along with demonstrating its antioxidant capacity and potential antibacterial properties. The subsequent experiments conducted involving graded alcohol extraction then identified the precipitate from the crude extract, with 70% alcohol exhibiting the strongest anti-cancer activity. Through the use of diverse GC-MS detection methods, we identified potential bioactive compounds with anti-cancer properties, notably sterol derivatives instead of conventional anti-cancer substances. Additionally, we performed a comparative analysis of the six different HE varieties, revealing significant variations between them. Our future research endeavors will primarily concentrate on unraveling the specific bioactive compounds responsible for the anti-cancer effects of HE and elucidating their underlying mechanisms of action.

## Figures and Tables

**Table 1:** Response surface design and results.

Treatment	A: Extracting Time (min)	B: Extracting Pressure (MPa)	C: Extracting Temperature (°C)	Extraction Rate (%)
1	60	34	35	2.14
2	60	26	35	1.86
3	80	30	35	2.48
4	100	30	37	2.23
5	60	30	33	2.05
6	100	30	33	2.04
7	80	34	37	2.30
8	80	30	35	2.50
9	60	30	37	2.15
10	100	26	35	1.99
11	80	30	35	2.63
12	80	34	33	2.21
13	100	34	35	2.11
14	80	30	35	2.37
15	80	26	33	1.69
16	80	30	35	2.56
17	80	26	37	2.25

**Table 2:** Analysis of variance results.

Source	Squares	Freedom	Mean Square	*F* Ratio	*p* Ratio
Model	0.9400	9	0.1044	12.4400	0.0016
A—Extracting time	0.0033	1	0.0033	0.3967	0.5488
B—Extracting pressure	0.1161	1	0.1161	13.8300	0.0075
C—Extracting temperature	0.1076	1	0.1076	12.8300	0.0090
AB	0.0064	1	0.0064	0.7598	0.4132
AC	0.0017	1	0.0017	0.2013	0.6673
BC	0.0552	1	0.0552	6.5800	0.0373
A^2^	0.2342	1	0.2342	27.9000	0.0011
B^2^	0.2530	1	0.2530	30.1400	0.0009
C^2^	0.0974	1	0.0974	11.6100	0.0113
Residual	0.0588	7	0.0084	-	-
Misfit term	0.0204	3	0.0068	0.7087	0.5953
Pure error	0.0384	4	0.0096	-	-
Total	0.9988	16	-	-	-

*R*^2^ = 0.9412, *R*^2^_adj_ = 0.8655.

**Table 3:** Response surface validation experiment results.

Numbered	Extraction Rate (%)	Mean Value (%)	RSD (%)
1	2.51	2.51	2.68
2	2.44		
3	2.57		

**Table 4:** Results of the model generality verification.

Variety	Extraction Rate Mean Value (%)	RSD (%)
He3	1.86	2.87
He13	1.88	3.97
T3	2.79	0.32
Gt	2.35	3.03
4916	2.62	4.78

**Table 5:** Antibacterial activity of the supercritical crude extracts of HE.

Experimental Design	*E*. *coli*	*S*. *aureus*	*B*. *subtilis*	*S*. *cerevisiae*
Deionized water	IH	\	\	\
Positive control	21.22 ± 0.13	19.71 ± 0.15	24.12 ± 0.21	18.69 ± 0.26
Absolute ethanol	\	\	\	\
1 mg/mL SFE crude extract	\	\	\	\
10 mg/mL SFE crude extract	\	17.35 ± 0.34	\	\
25 mg/mL SFE crude extract	18.54 ± 0.21	\	\	\

IH: inhibition zone with bacterial growth inside. \: no inhibition zone or an inhibition zone smaller than 9 mm was observed.

**Table 6:** Material characteristics.

Project	Parameter	Project	Parameter
Moisture content	10%	Bacteria number × 1	<1 × 10^2^ CFU/g
Density	1.9	Mold spore × 2	<1 × 10^1^ CFU/g
Particle size	0.85 mm	Impurity × 3	<1 mg/g

**Table 7:** Factor analysis and horizontal design of the response surface method.

Factor	Unit	Low Levels (−1)	High Levels (+1)
A: Extracting time	min	60	100
B: Extracting pressure	MPa	26	34
C: Extracting temperature	°C	33	37

## Data Availability

The data used to support the findings of this study are available from the corresponding author (Xiaoming Chen, chxm001@126.com) upon request.

## Supplementary Materials

The following supporting information can be downloaded at: https://www.mdpi.com/article/10.3390/molecules28176288/s1, GC-MS composition table of supercritical crude extracts of six varieties of HE: Table S1: 4916; Table S2: Gt; Table S3: He3; Table S4: He12; Table S5: He13; Table S6: T3.

## Sample Availability

Samples of the HE used in this study are available from the authors.

## References

[1] Rodrigues D.M., Freitas A.C., Rocha-Santos T.A., Vasconcelos M.W., Roriz M., Rodriguez-Alcala L.M., Duarte A.C. (2015). Chemical composition and nutritive value of *Pleurotus citrinopileatus* var *cornucopiae*, *P. eryngii*, *P. salmoneo stramineus*, *Pholiota nameko* and *Hericium erinaceus*. J. Food Sci. Technol..

[2] Friedman M. (2015). Chemistry, nutrition, and health-promoting properties of *Hericium erinaceus* (lion’s mane) mushroom fruiting bodies and mycelia and their bioactive compounds. J. Agric. Food Chem..

[3] Komura D.L., Ruthes A.C., Carbonero E.R., Gorin P.A., Iacomini M. (2014). Water-soluble polysaccharides from *Pleurotus ostreatus* var. *florida* mycelial biomass. Int. J. Biol. Macromol..

[4] Rossi P., Cesaroni V., Brandalise F., Occhinegro A., Ratto D., Perrucci F., Savino E. (2018). Dietary supplementation of lion’s mane medicinal mushroom, *Hericium erinaceus* (Agaricomycetes), and spatial memory in wild-type mice. Int. J. Med. Mushrooms.

[5] Wang X.Y., Zhang D.D., Yin J.Y., Nie S.P., Xie M.Y. (2019). Recent developments in *Hericium erinaceus* polysaccharides: Extraction, purification, structural characteristics and biological activities. Crit. Rev. Food Sci. Nutr..

[6] Silva A.D., Sartori D., Macedo F.C., Ribeiro L.R., Fungaro M.H., Mantovani M.S. (2013). Effects of β-glucan extracted from *Agaricus blazei* on the expression of ERCC5, CASP9, and CYP1A1 genes and metabolic profile in HepG2 cells. Hum. Exp. Toxicol..

[7] Wong K.H., Naidu M., David P., Abdulla M.A., Abdullah N., Kuppusamy U.R., Sabaratnam V. (2011). Peripheral nerve regeneration following crush injury to rat peroneal nerve by aqueous extract of medicinal mushroom *Hericium erinaceus* (Bull.: Fr) Pers. (Aphyllophoromycetideae). Evid.-Based Complement. Altern. Med..

[8] Zou P., Guo Y., Ding S., Song Z., Cui H., Zhang Y., Chen X. (2022). Autotoxicity of Endogenous Organic Acid Stress in Two *Ganoderma lucidum* Cultivars. Molecules.

[9] Wang B.H., Cao J.J., Zhang B., Chen H.Q. (2019). Structural characterization, physicochemical properties and α-glucosidase inhibitory activity of polysaccharide from the fruits of wax apple. Carbohydr. Polym..

[10] Spigno G., De Faveri D.M. (2007). Antioxidants from grape stalks and marc: Influence of extraction procedure on yield, purity and antioxidant power of the extracts. J. Food Eng..

[11] Vagi E., Simandi B., Hethelyi E. (2005). Essential oil composition and antimicrobial activity of *Origanum majorana L* extracts obtained with ethyl alcohol and supercritical carbon dioxide. Food Res. Int..

[12] Benito-Roman O., Alonso E., Cocero M.J., Goto M. (2016). β-Glucan recovery from *Ganoderma lucidum* by means of pressurized hot water and supercritical CO_2_. Food Bioprod. Process..

[13] Jaferian S., Negahdari B., Eatemadi A. (2016). Colon cancer targeting using conjugates biomaterial 5-flurouracil. Biomed. Pharmacother..

[14] Arnold M., Sierra M.S., Laversanne M., Soerjomataram I., Jemal A., Bray F. (2017). Global patterns and trends in colorectal cancer incidence and mortality. Gut.

[15] Miller K.D., Nogueira L., Mariotto A.B., Rowland J.H., Yabroff K.R., Alfano C.M., Siegel R.L. (2019). Cancer treatment and survivorship statistics. Cancer Treat. Surviv. Stat..

[16] Siegel R.L., Torre L.A., Soerjomataram I., Hayes R.B., Bray F., Weber T.K., Jemal A. (2019). Global patterns and trends in colorectal cancer incidence in young adults. Gut.

[17] McWhirter D., Kitteringham N., Jones R.P., Malik H., Park K., Palmer D. (2013). Chemotherapy induced hepatotoxicity in metastatic colorectal cancer: A review of mechanisms and outcomes. Crit. Rev. Oncol./Hematol..

[18] Berna A., Chafer A., Monton J.B. (2001). High-pressure solubility data of the system resveratrol (3) + ethanol (2) + CO_2_ (1). J. Supercrit. Fluids.

[19] Pascual-Marti M.C., Salvador A., Chafer A., Berna A. (2001). Supercritical fluid extraction of resveratrol from grape skin of Vitis vinifera and determination by HPLC. Talanta.

[20] Benova B., Adam M., Pavlikova P., Fischer J. (2010). Supercritical fluid extraction of piceid, resveratrol and emodin from Japanese knotweed. J. Supercrit. Fluids.

[21] Casas L., Mantell C., Rodriguez M., Roldan A., De Ory I., Blandino A. (2010). Extraction of resveratrol from the pomace of Palomino fino grapes by supercritical carbon dioxide. J. Food Eng..

[22] Shrigod N.M., Swami Hulle N.R., Prasad R.V. (2016). Supercritical fluid extraction of essential oil from mint leaves (mentha spicata): Process optimization and its quality evaluation. J. Food Process Eng..

[23] Ara K.M., Jowkarderis M., Raofie F. (2015). Optimization of supercritical fluid extraction of essential oils and fatty acids from flixweed (*Descurainia sophia* L.) seed using response surface methodology and central composite design. J. Food Sci. Technol..

[24] Guan W., Li S., Yan R., Tang S., Quan C. (2007). Comparison of essential oils of clove buds extracted with supercritical carbon dioxide and other three traditional extraction methods. Food Chem..

[25] Ansari K., Goodarznia I. (2012). Optimization of supercritical carbon dioxide extraction of essential oil from spearmint (*Mentha spicata* L.) leaves by using Taguchi methodology. J. Supercrit. Fluids.

[26] Yothipitak W., Thana P., Goto M., Shotipruk A. (2008). Experiments and statistical analysis of supercritical carbon dioxide extraction. Chiang Mai J. Sci..

[27] Jitrangsri K., Chaidedgumjorn A., Satiraphan M. (2020). Supercritical fluid extraction (SFE) optimization of trans-resveratrol from peanut kernels (*Arachis hypogaea*) by experimental design. J. Food Sci. Technol..

[28] Liu W., Fu Y.J., Zu Y.G., Tong M.H., Wu N., Liu X.L., Zhang S. (2009). Supercritical carbon dioxide extraction of seed oil from Opuntia dillenii Haw and its antioxidant activity. Food Chem..

[29] Mensor L.L., Menezes F.S., Leitao G.G., Reis A.S., Santos T.C., Coube C.S., Leitao S.G. (2001). Screening of Brazilian plant extracts for antioxidant activity by the use of DPPH free radical method. Phytother. Res..

[30] Chong P.S., Poon C.H., Roy J., Tsui K.C., Lew S.Y., Phang M.W., Lim L.W. (2021). Neurogenesis-dependent antidepressant-like activity of *Hericium erinaceus* in an animal model of depression. Chin. Med..

[31] Kitzberger C.S., Smania A., Pedrosa R.C., Ferreira S.R.S. (2007). Antioxidant and antimicrobial activities of shiitake (*Lentinula edodes*) extracts obtained by organic solvents and supercritical fluids. J. Food Eng..

[32] Smania A., Smania E.F., Monache F.D., Pizzolatti M.G., Monache G.D. (2006). Derivatization does not influence antimicrobial and antifungal activities of applanoxidic acids and sterols from *Ganoderma* spp.. Z. Fur Naturforschung C.

[33] Smania A., Monache F.D., Smania E.D., Cuneo R.S. (1999). Antibacterial activity of steroidal compounds isolated from *Ganoderma applanatum* (Pers.) Pat. (Aphyllophoromycetideae) fruit body. Int. J. Med. Mushrooms.

[34] Sahu P.K., Ramisetti N.R., Cecchi T., Swain S., Patro C.S., Panda J. (2017). An overview of experimental designs in HPLC method development and validation. J. Pharm. Biomed. Anal..

[35] Koh G.Y., Chou G., Liu Z. (2009). Purification of a water extract of Chinese sweet tea plant (*Rubus suavissimus* s. lee) by alcohol precipitation. J. Agric. Food Chem..

[36] Schmourlo G., Mendonca-Filho R.R., Alviano C.S., Costa S.S. (2005). Screening of antifungal agents using ethanol precipitation and bioautography of medicinal and food plants. J. Ethnopharmacol..

