# Supplementary material for: Enrichment Extraction and Activity Study of the Different Varieties of Hericium erinaceus against HCT-8 Colon Cancer Cells
Source: Molecules. 2023 Aug 28;28(17):6288. doi: 10.3390/molecules28176288 (PMC10488996; doi:10.3390/molecules28176288)
Supplement: Supplementary file 1 [file molecules-28-06288-s001.zip › molecules-2538706-supplementary.pdf]

Supplementary Materials

Table S1. 4916

peak number	retention time（min）	peak area	peak area（%）	extraction rate（%）
1	6.895	486764	0.01	1-Butanol, 3-methyl-
2	9.050	342889	0.01	2-Propanone, 1-hydroxy-
3	9.825	368429	0.01	5-Hepten-2-one, 6-methyl-
4	11.358	270713	0.00	2-Nonen-1-ol
5	11.531	285839	0.01	7-Oxabicyclo [2.2.1] heptane
6	12.670	3727777	0.07	Acetic acid
7	13.057	296581	0.01	Furfural
8	14.145	285142	0.01	Formic acid
9	15.869	108000	0.00	2-Furancarboxaldehyde, 5-methyl-
10	16.110	1483783	0.03	2-Pentadecanol
11	16.265	65194	0.00	2-Undecanone
12	17.735	213756	0.00	Pentanoic acid, heptyl ester
13	22.010	872764	0.02	Hexanoic acid
14	24.380	999930	0.02	1,3-Benzenediol, 2-methyl-
15	25.674	3342850	0.06	Tetrahydro-4H-pyran-4-ol
16	25.920	1774002	0.03	1-Heptyn-6-one
17	26.071	4719299	0.08	Isopropyl myristate
18	27.518	1199269	0.02	3-Methyl-3-oxetanemethanol
19	28.189	971136	0.02	1,4-Cyclohexanediol, trans-
20	28.703	576851	0.01	1,8(2H,5H)-Naphthalenedione, hexahydro-8a-methyl-, cis-
21	28.782	1823830	0.03	Nonanoic acid
22	29.221	526649	0.01	l-Pantoyl lactone
23	29.385	1249461	0.02	2-Hepten-4-one, 2-methyl-
24	29.913	1543446	0.03	Hexadecanoic acid, methyl ester
25	30.635	18101478	0.32	Hexadecanoic acid, ethyl ester
26	31.665	31583898	0.56	Glycerin
27	31.808	4989145	0.09	Pentanoic acid, 4-oxo-
28	33.813	2685663	0.05	10-Octadecenoic acid, methyl ester
29	34.049	5406416	0.10	Pentadecanoic acid, ethyl ester
30	34.147	2729895	0.05	2-Pentenoic acid, 4,4-dimethyl-, methyl ester
31	34.340	16056230	0.29	(E)-9-Octadecenoic acid ethyl ester
32	34.457	1371217	0.02	9-Octadecenoic acid, ethyl ester
33	34.533	3550749	0.06	9,12-Octadecadienoic acid, methyl ester
34	35.039	26070804	0.47	9,12-Octadecadienoic acid, ethyl ester
35	35.331	1624682	0.03	Benzeneacetic acid

36	35.616	17666962	0.32	4-(Hydroxymethyl)-3-oxabicyclo [3.1.0] hexan-2-one
37	37.181	29171965	0.52	Tridecanoic acid
38	38.042	4013191	0.07	Linoelaidic acid
39	38.515	165661240	2.96	Pentadecanoic acid
40	40.167	1123044609	20.04	n-Hexadecanoic acid
41	40.578	52545417	0.94	Cyclopentadecanone, 2-hydroxy-
42	41.555	40566705	0.72	cis-10-Heptadecenoic acid
43	41.884	88848804	1.59	1,2,3,4-Butanetetrol, [S- (R*, R*)]-
44	42.769	6645340	0.12	2,5-Monoformal-l-rhamnitol
45	44.071	535422855	9.55	Octadecanoic acid
46	44.819	1581105313	28.21	9-Octadecenoic acid
47	46.280	1818617729	32.45	9,12-Octadecadienoic acid (Z, Z)-

Table S2. Gt

peak number	retention time（min）	peak area	peak area（%）	extraction rate（%）
1	6.881	347209	0.01	1-Butanol, 3-methyl-
2	9.018	365272	0.01	2-Propanone, 1-hydroxy-
3	9.827	925557	0.01	5-Hepten-2-one, 6-methyl-
4	11.369	248029	0.00	2-Octene, 2-methyl-6-methylene-
5	11.503	452093	0.01	2(5H)-Furanone, 5-methyl-
6	12.641	4417068	0.06	Acetic acid
7	13.044	282391	0.00	Furfural
8	14.092	547143	0.01	Formic acid
9	14.809	400778	0.01	Ethyl formate
10	15.808	358455	0.01	2-Furancarboxaldehyde, 5-methyl-
11	16.033	614483	0.01	4-Cyclopentene-1,3-dione
12	16.068	879898	0.01	Propylene Glycol
13	16.269	265314	0.00	2-Cyclopenten-1-one, 4-methoxy-
14	17.064	1858121	0.03	Butyrolactone
15	17.708	822833	0.01	2-Furanmethanol
16	17.955	1633558	0.02	Butanoic acid, 3-methyl-
17	19.790	424748	0.01	Heptasiloxane, hexadecamethyl-
18	21.966	1864352	0.03	Hexanoic acid
19	24.335	1934804	0.03	1,3-Benzenediol, 2-methyl-
20	25.624	10054907	0.15	2-Deoxy-2-fluoro-1,6-anhydro-beta-d-glucopyranose
21	25.868	1533625	0.02	2-Hexanone, 5-methyl-
22	26.042	2707842	0.04	3-Octen-2-ol
23	27.472	1329496	0.02	1-Penten-3-ol, 4-methyl-

24	28.136	978594	0.01	1,4-Cyclohexanediol, trans-
25	28.668	1513906	0.02	1,8(2H,5H)-Naphthalenedione, hexahydro-8a-methyl-, cis-
26	28.750	2839852	0.04	Nonanoic acid
27	29.175	734714	0.01	Cyclopropanemethanol, alpha-butyl-
28	29.348	1412213	0.02	3-Decen-5-one
29	29.855	1067046	0.02	Hexadecanoic acid, methyl ester
30	30.596	36115307	0.53	Hexadecanoic acid, ethyl ester
31	31.674	64919996	0.95	Glycerin
32	33.781	2237877	0.03	7-Octadecenoic acid, methyl ester
33	34.030	9915890	0.15	Pentadecanoic acid, ethyl ester
34	34.114	3737379	0.05	2-Pentenoic acid, 4,4-dimethyl-, methyl ester
35	34.322	48362313	0.71	9-Octadecenoic acid, ethyl ester
36	34.435	5916338	0.09	(E)-9-Octadecenoic acid ethyl ester
37	34.505	2192788	0.03	9,12-Octadecadienoic acid, methyl ester
38	35.024	68417657	1.00	9,12-Octadecadienoic acid, ethyl ester
39	35.294	1836026	0.03	Benzeneacetic acid
40	35.578	28094111	0.41	1,2-Ethanediol, 1-(2-furanyl)-
41	37.153	43090866	0.63	Tetradecanoic acid
42	37.628	5178196	0.08	Fumaric acid, ethyl 2-methylallyl ester
43	38.052	6891512	0.10	Pentyl linoleate
44	38.490	210022458	3.08	Pentadecanoic acid
45	40.132	1426244091	20.92	n-Hexadecanoic acid
46	40.579	81777002	1.20	Cyclopentadecanone, 2-hydroxy-
47	41.524	42465957	0.62	cis-10-Heptadecenoic acid
48	41.951	171168240	2.51	1,2,3,4-Butanetetrol, [S- (R*, R*)]-
49	42.756	15852389	0.23	2,5-Monoformal-l-rhamnitol
50	44.099	676289827	9.92	Octadecanoic acid
51	44.840	1848316213	27.11	9-Octadecenoic acid
52	46.280	1974996898	28.97	9,12-Octadecadienoic acid (Z, Z)-

Table S3. He3

peak number	retention time（min）	peak area	peak area（%）	extraction rate（%）
1	12.679	828152	0.04	Acetic acid
2	16.100	268067	0.01	Propylene Glycol
3	22.002	975128	0.05	Heptanoic acid
4	24.306	285640	0.01	Octanoic acid
5	26.076	1156547	0.05	3-Octen-2-ol
6	27.508	361919	0.02	1-Penten-3-ol, 4-methyl-
7	28.158	436196	0.02	1,4-Cyclohexanediol, trans-
8	28.783	1529778	0.07	Nonanoic acid
9	29.426	570664	0.03	2-Hepten-4-one, 2-methyl-
10	29.936	1347940	0.06	Hexadecanoic acid, methyl ester
11	30.647	5409954	0.26	Hexadecanoic acid, ethyl ester
12	31.607	3346093	0.16	Glycerin
13	31.793	887368	0.04	Pentanoic acid, 4-oxo-
14	32.113	540937	0.03	1,3-Propanediol, 2-ethyl-2-(hydroxymethyl)-
15	34.032	1114184	0.05	Pentadecanoic acid, ethyl ester
16	34.130	528165	0.02	2-Pentenoic acid, 4,4-dimethyl-, methyl ester
17	34.322	4990194	0.24	(E)-9-Octadecenoic acid ethyl ester
18	34.519	2688820	0.13	9,12-Octadecadienoic acid, methyl ester
19	35.017	6887708	0.33	9,12-Octadecadienoic acid, ethyl ester
20	35.311	426563	0.02	Benzeneacetic acid
21	35.597	2614355	0.12	4-(Hydroxymethyl)-3-oxabicyclo [3.1.0] hexan-2-one
22	37.181	9620612	0.45	Benzene, 1,1'-[1,2-ethanediylbis(oxy)]bis-
23	37.645	1882419	0.09	Fumaric acid, ethyl 2-methylallyl ester
24	38.473	63564412	3.00	Pentadecanoic acid
25	40.018	544555993	25.70	n-Hexadecanoic acid
26	40.489	15083934	0.71	9-Hexadecenoic acid
27	41.510	2117232	0.10	6-Octadecenoic acid, (Z)-
28	41.602	4221377	0.20	Heptadecanoic acid
29	43.754	126126175	5.95	Octadecanoic acid
30	44.486	545454110	25.74	6-Octadecenoic acid, (Z)-
31	45.875	769222734	36.30	9,12-Octadecadienoic acid (Z, Z)-

Table S4. He12

peak number	retention time（min）	peak area	peak area（%）	extraction rate（%）
1	6.874	90581	0.00	1-Butanol, 3-methyl-
2	12.670	1788929	0.04	Acetic acid
3	16.077	433468	0.01	4-Penten-2-ol
4	17.097	747763	0.02	Butyrolactone
5	17.915	428410	0.01	Butanoic acid, 3-methyl-
6	21.988	1098910	0.02	Hexanoic acid
7	25.648	1002646	0.02	2-Deoxy-2-fluoro-1,6-anhydro-beta-d-glucopyranose
8	25.887	2324758	0.05	2-Hexanone, 5-methyl-
9	26.055	2239833	0.05	3-Octen-2-ol
10	26.872	462137	0.01	3-Octen-2-ol
11	27.495	857627	0.02	3-Methyl-3-oxetanemethanol
12	27.907	653030	0.01	Pentanal, 3-hydroxy-2-methyl-
13	28.156	728900	0.02	1,4-Cyclohexanediol, trans-
14	28.755	3674673	0.08	Nonanoic acid
15	29.183	755787	0.02	Cyclopropanemethanol, alpha-butyl-
16	29.394	1169456	0.03	3-Decen-5-one
17	29.916	1014995	0.02	Hexadecanoic acid, methyl ester
18	30.629	8647954	0.19	Hexadecanoic acid, ethyl ester
19	31.683	2647701	0.06	Glycerin
20	31.790	1589860	0.04	Pentanoic acid, 4-oxo-
21	33.796	2848133	0.06	10-Octadecenoic acid, methyl ester
22	34.030	2077386	0.05	Pentadecanoic acid, ethyl ester
23	34.324	15079909	0.33	9-Octadecenoic acid, ethyl ester
24	34.446	1391849	0.03	(E)-9-Octadecenoic acid ethyl ester
25	34.518	2311661	0.05	9,12-Octadecadienoic acid, methyl ester
26	35.022	17958824	0.40	9,12-Octadecadienoic acid, ethyl ester
27	35.301	2310193	0.05	Benzeneacetic acid
28	35.595	4008107	0.09	4-(Hydroxymethyl)-3-oxabicyclo [3.1.0] hexan-2-one
29	37.175	28045996	0.62	Benzene, 1,1'-[1,2-ethanediylbis(oxy)]bis-
30	37.633	3045988	0.07	Fumaric acid, 2,4-dimethylpent-3-yl ethyl ester
31	38.005	5243457	0.12	Isopropyl linoleate
32	38.473	106685682	2.36	Pentadecanoic acid
33	40.082	836370806	18.48	n-Hexadecanoic acid
34	40.509	28879767	0.64	cis-10-Heptadecenoic acid
35	41.600	19922236	0.44	Heptadecanoic acid

36	43.933	349856077	7.73	Octadecanoic acid
37	44.724	1499091483	33.13	6-Octadecenoic acid, (Z)-
38	46.119	1567152196	34.64	9,12-Octadecadienoic acid (Z, Z)-

Table S5. He13

peak number	retention time（min）	peak area	peak area（%）	extraction rate（%）
1	6.820	72213	0.00	2-Pentene
2	8.986	236891	0.01	2-Propanone, 1-hydroxy-
3	9.798	146978	0.00	5-Hepten-2-one, 6-methyl-
4	9.873	105628	0.00	Propanoic acid, 2-hydroxy-, ethyl ester, (L)-
5	11.511	254992	0.01	2-Carene
6	12.662	1611773	0.04	Acetic acid
7	14.142	448057	0.01	Formic acid
8	16.092	249336	0.01	R-(-)-1,2-propanediol
9	17.089	609995	0.02	Butyrolactone
10	21.996	547774	0.01	Hexanoic acid
11	24.362	474672	0.01	1,3-Benzenediol, 2-methyl-
12	25.540	227816	0.01	Octanoic acid, octyl ester
13	25.652	304545	0.01	1,3-Pentanediol, 2,2,4-trimethyl-
14	25.899	625105	0.02	1-Heptyn-6-one
15	26.074	5308535	0.14	Isopropyl myristate
16	27.505	765147	0.02	3-Methyl-3-oxetanemethanol
17	28.165	614823	0.02	1,4-Cyclohexanediol, trans-
18	28.603	817170	0.02	Pentadecanoic acid, ethyl ester
19	28.756	1244215	0.03	Nonanoic acid
20	29.404	821028	0.02	3-Decen-5-one
21	29.911	754785	0.02	Hexadecanoic acid, methyl ester
22	30.628	8861917	0.23	Hexadecanoic acid, ethyl ester
23	31.620	11098162	0.29	Glycerin
24	33.795	1597819	0.04	10-Octadecenoic acid, methyl ester
25	34.029	2388520	0.06	Pentadecanoic acid, ethyl ester
26	34.128	2744436	0.07	2-Pentenoic acid, 4,4-dimethyl-, methyl ester
27	34.324	11270695	0.29	9-Octadecenoic acid, ethyl ester
28	34.444	422258	0.01	(E)-9-Octadecenoic acid ethyl ester
29	34.516	2415626	0.06	9,12-Octadecadienoic acid, methyl ester
30	35.021	19374968	0.51	9,12-Octadecadienoic acid, ethyl ester
31	35.311	859239	0.02	Benzeneacetic acid
32	35.592	13053460	0.34	2-Pentenoic acid, 3-methyl-, methyl ester

33	37.188	23385228	0.61	Benzene, 1,1'-[1,2-ethanediylbis(oxy)]bis-
34	37.650	2759931	0.07	Fumaric acid, ethyl 2-methylallyl ester
35	37.999	1316974	0.03	Pentyl linoleate
36	38.485	111700001	2.91	Pentadecanoic acid
37	40.089	841848422	21.97	n-Hexadecanoic acid
38	40.515	25703556	0.67	9-Eicosenoic acid, (Z)-
39	41.505	12910682	0.34	Palmitoleic acid
40	43.876	277427337	7.24	Octadecanoic acid
41	44.634	1026372386	26.78	9-Octadecenoic acid
42	46.075	1418554073	37.02	9,12-Octadecadienoic acid (Z, Z)-

Table S6. T3

peak number	retention time（min）	peak area	peak area（%）	extraction rate（%）
1	6.740	316956	0.01	1-Butanol, 3-methyl-
2	12.641	2119383	0.07	Acetic acid
3	16.079	245363	0.01	R-(-)-1,2-propanediol
4	16.226	345587	0.01	Dimethyl Sulfoxide
5	17.107	297864	0.01	Butanoic acid, 4-hydroxy-
6	17.916	296101	0.01	Butanoic acid, 3-methyl-
7	21.988	944381	0.03	Hexanoic acid
8	24.302	329613	0.01	Heptanoic acid
9	25.891	1764370	0.06	2-Hexanone, 4-hydroxy-5-methyl-3-propyl-
10	26.059	1322547	0.04	3-Octen-2-ol
11	27.490	481736	0.02	1-Penten-3-ol, 4-methyl-
12	28.156	538430	0.02	1,4-Cyclohexanediol, trans-
13	28.755	2907563	0.09	Nonanoic acid
14	29.193	515262	0.02	Cyclopropanemethanol, alpha-butyl-
15	29.394	1165265	0.04	2-Hepten-4-one, 2-methyl-
16	29.918	1426180	0.04	Hexadecanoic acid, methyl ester
17	30.634	11045514	0.35	Hexadecanoic acid, ethyl ester
18	31.625	7228173	0.23	Glycerin
19	31.784	2632259	0.08	Pentanoic acid, 4-oxo-
20	33.796	1877955	0.06	9-Octadecenoic acid, methyl ester
21	34.031	2338725	0.07	Pentadecanoic acid, ethyl ester
22	34.127	1150014	0.04	Tetrahydrofuran, 2-ethyl-5-butyl-
23	34.321	15836541	0.50	9-Octadecenoic acid, ethyl ester
24	34.441	1176772	0.04	(E)-9-Octadecenoic acid ethyl ester

25	34.520	2707044	0.09	9,12-Octadecadienoic acid, methyl ester
26	35.019	21241814	0.67	9,12-Octadecadienoic acid, ethyl ester
27	35.303	1419645	0.04	Benzeneacetic acid
28	35.589	3929671	0.12	2-Pentenoic acid, 3-methyl-, methyl ester
29	37.157	11811116	0.37	Tetradecanoic acid
30	37.640	2960807	0.09	Fumaric acid, ethyl 2-methylallyl ester
31	37.996	3884578	0.12	E, Z-2,13-Octadecadien-1-ol acetate
32	38.470	77243538	2.43	Pentadecanoic acid
33	40.048	680111990	21.40	n-Hexadecanoic acid
34	40.496	24093068	0.76	9-Hexadecenoic acid
35	41.509	13216611	0.42	cis-10-Nonadecenoic acid
36	43.822	220917785	6.95	Octadecanoic acid
37	44.596	952243756	29.97	9-Octadecenoic acid
38	45.980	1103326653	34.72	9,12-Octadecadienoic acid (Z, Z)-
